# Endoscopic salvage of a *N* -butyl cyanoacrylate-occluded gastroscope using cholangioscopic electrohydraulic lithotripsy

**DOI:** 10.1055/a-2895-4914

**Published:** 2026-07-16

**Authors:** Sengwang Fu, Yuqing Mao, Bin Shi

**Affiliations:** 1 Digestive Endoscopy CenterYueyang Hospital of Integrated Traditional Chinese and Western Medicine, Shanghai University of Traditional Chinese MedicineShanghaiChina; 2 Department of Gastroenterology 12482 Shanghai General HospitalShanghaiChina

A 57-year-old man with decompensated cirrhosis was admitted for acute hemorrhage from esophagogastric varices. During endoscopic hemostasis, intra-variceal injection of *N* -butyl cyanoacrylate (NBCA) was administered to gastric fundal varices. Immediately following injection, NBCA polymerized upon contact with a luminal fluid and refluxed accidentally into the working channel, resulting in the complete obstruction of the therapeutic gastroscope. The occluded scope was withdrawn and exchanged for a new one to complete the hemostatic procedure, as the original was rendered nonfunctional.

**Fig. 1:**
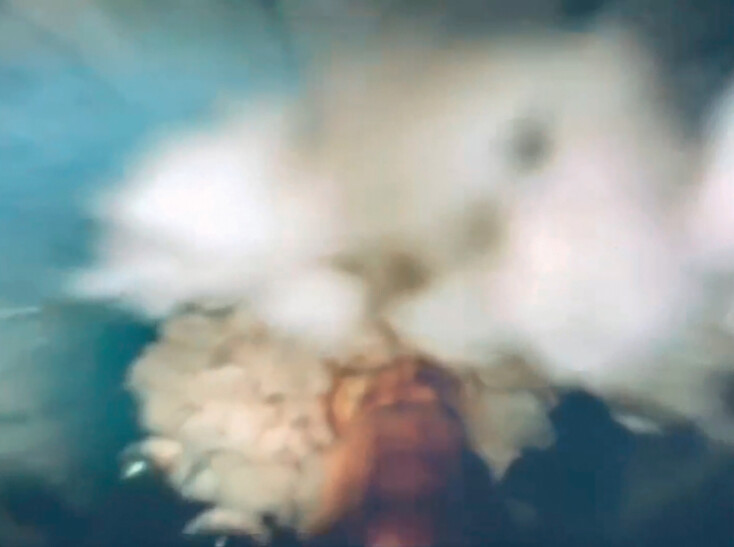
Direct cholangioscope vision revealed large NBCA particles obstructing the lumen, with an electrohydraulic probe targeting them.

**Fig. 2:**
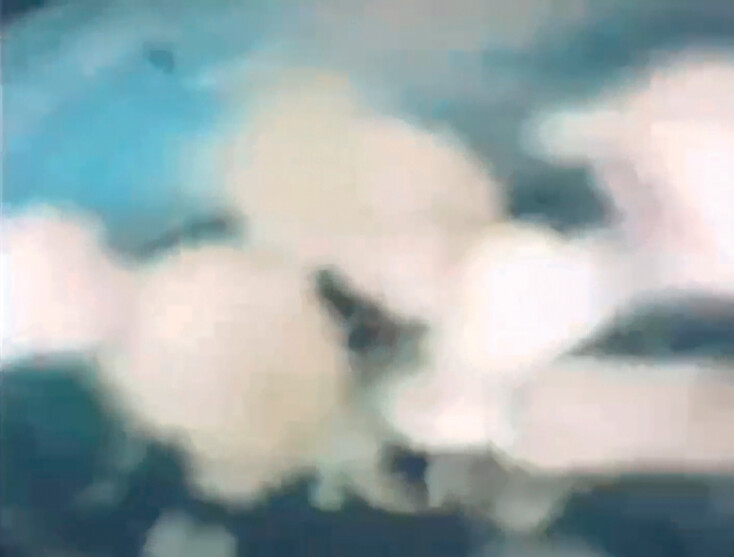
The NBCA particles were progressively fragmented into small particles by cholangioscopic electrohydraulic lithotripsy.

**Fig. 3:**
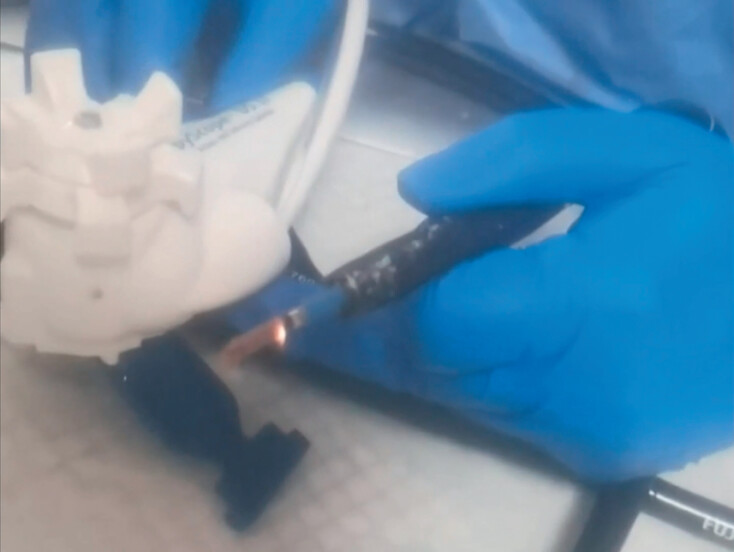
The NBCA particles were then pushed out via the working channel of the gastroscope.

**Fig. 4:**
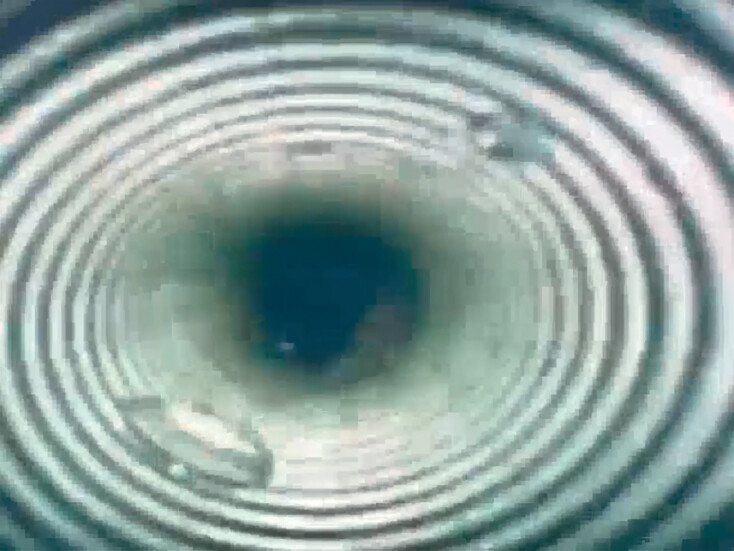
Post-procedural inspection confirmed the complete clearance of the working channel without macroscopic damage to its lining.

Standard salvage maneuvers, including high-pressure water irrigation, disposable brushes, and alligator forceps, were used to remove the hardened NBCA of the working channel but failed. The NBCA had solidified and tenaciously adhered to the channel wall, rendering conventional methods ineffective. Therefore, we attempted to use a training cholangioscope to fragment the solidified NBCA via lithotripsy. Direct cholangioscope vision revealed large NBCA casts obstructing the lumen, with an electrohydraulic probe targeting them ( Fig. 1 ). Electrohydraulic lithotripsy (EHL) was performed at an energy set of 0.3J per pulse, with continuous irrigation of sterile distilled water at 5 mL/min. The NBCA particles were progressively fragmented into small particles ( Fig. 2 ), which were then pushed out ( Fig. 3 , Video 1 ). Post-procedural inspection confirmed the complete clearance of the working channel without macroscopic damage to its lining ( Fig. 4 ).

**Video 1** Endoscopic salvage of a *N* -butyl cyanoacrylate-occluded gastroscope using cholangioscopic electrohydraulic lithotripsy.


NBCA is highly effective for variceal obliteration 1​ ​2​ ; however, intraluminal polymerization cause critical damage to the endoscope, leading to substantial repair costs or prolonged downtime. 3​ Conventional mechanical or hydraulic methods are often inadequate for large, solidified NBCA casts. This case demonstrates that cholangioscopic EHL provides a safe, efficient, and cost-effective salvage strategy, potentially obviating the need for costly factory repair and prolonged instrument unavailability.

Endoscopy_UCTN_Code_TTT_1AO_2AO
